# Supplementary material for: Recognition of the game situation in baseball
Source: PLoS One. 2024 Aug 20;19(8):e0309328. doi: 10.1371/journal.pone.0309328 (PMC11335100; doi:10.1371/journal.pone.0309328)
Supplement: Attachment — Submitted filename: Submissions Needing Revision.docx [file pone.0309328.s001.docx]

**Response Letter**

Reviewer #1: The statistical analysis in this manuscript was fascinating. I found it interesting that the author chose a hypothetical baseball game with a score of 0-0 in the 1st inning. Baseball seems to have an infinite amount of situations, but the author did a great job to break down a fraction of them and draw conclusions. Great work!

**Response:** Thank you very much. We are extremely grateful for your comments on our manuscript.

Reviewer #2: The survey is thorough, well analyzed, and has an interesting premise. However, the scope is limited in many ways. The context of a first inning, no 0-0 game score is, itself, a factor in judging pitcher or batter advantage. How different might the results be in contexts such as late innings with "your" team behind (or ahead) by one run? It is meaningful that hitters have more extreme views of pitch count advantage for both batters and pitchers. Of course, batters have much more uncertainty.

**Response:** As a limitation of this study, we did not take into account some game situations and factors, such as the inning (early, middle, or late), top or bottom of the inning, number of pitches per pitcher, score, or home or away games. For example, a previous research showed that the weighting in the probability of winning differed between the first and ninth innings [Lindsey, 1961]. In addition, other factors such as the batter's good or bad performance may be related to the recognition of actual baseball games. These points are listed as limitations (page 18-19, lines 324-329):

“Second, we did not take into account some game situations and factors, such as the inning (early, middle, or late), top or bottom of the inning, number of pitches per pitcher, score, or home or away games. For example, research showed that the weighting in the probability of winning differed between the first and ninth innings [1]. Additionally, other factors such as the batter's good or bad performance may be related to the recognition of actual baseball games.”

Additional Editor Comments:

Two reviewers have assessed the paper. While both reviews were generally positive, there is concern over the very narrow scope of the situation assessed in the paper. Please address the potential (or lack thereof) for obtaining different results depending on the situation (eg. inning and score) in the game. Reviewer #2 also discussed the idea that uncertainty may be higher for batters than for pitchers. Please discuss this point. That is, do the authors agree with this point and how is this idea potentially reflected in

**Response:** We appreciate this constructive comment. We added the discussion (pages 18, lines 309-313):

“However, we also considered that our data could not apply to and fit all cases in the game situation. For example, when the score is tied, the importance of performance increases in the ninth inning than in the first inning [1]. This may lead batters to be more sensitive about their perception of the count, but pitchers may also show a similar tendency.”

In addition, we added an explanation for the reason in greater cognitive changes in batters than pitchers (pages 18, lines 313-318):

“Additionally, the difference in the number of plays might be associated with the difference in perception between batters and pitchers. That is, once a batter is in at-bat, there is an interval of eight batters before the next at-bat as batting order. In contrast, a pitcher faces all batters continuously. The small number of batting play in a baseball game may lead batters to be more sensitive.”

Additional requirements:

Please review your reference list to ensure that it is complete and correct. If you have cited papers that have been retracted, please include the rationale for doing so in the manuscript text, or remove these references and replace them with relevant current references. Any changes to the reference list should be mentioned in the rebuttal letter that accompanies your revised manuscript. If you need to cite a retracted article, indicate the article’s retracted status in the References list and also include a citation and full reference for the retraction notice.

**Response:** The address of the reference in citation No. 23 was incomplete. Therefore, we have corrected it to include the complete address (page 24, lines 413-416).

“[23] Une N. The "most average hitter" in 2021 is... The average OPS of players who have reached the regular at-bat is .783 for Central League and .763 for Pacific League. [in Japanese; Internet]. 2021; <https://news.yahoo.co.jp/byline/unenatsuki/20211209->00271866. [Accessed 2 May 2023].”

If applicable, we recommend that you deposit your laboratory protocols in protocols.io to enhance the reproducibility of your results. Protocols.io assigns your protocol its own identifier (DOI) so that it can be cited independently in the future. For instructions see: https://journals.plos.org/plosone/s/submission-guidelines#loc-laboratory-protocols. Additionally, PLOS ONE offers an option for publishing peer-reviewed Lab Protocol articles, which describe protocols hosted on protocols.io. Read more information on sharing protocols at https://plos.org/protocols?utm_medium=editorial-email&utm_source=authorletters&utm_campaign=protocols.

**Response:** All relevant data and laboratory protocols are available from the corresponding author on reasonable request (page 24, lines 411-414).

The manuscript has now been rechecked by a native English speaker.
