# Supplementary material for: Recognition of the game situation in baseball
Source: PLoS One. 2024 Aug 20;19(8):e0309328. doi: 10.1371/journal.pone.0309328 (PMC11335100; doi:10.1371/journal.pone.0309328)
Supplement: Attachment — Submitted filename: Submissions Needing Revision2.docx [file pone.0309328.s002.docx]

**Response Letter**

**Journal Requirements:**

Please review your reference list to ensure that it is complete and correct. If you have cited papers that have been retracted, please include the rationale for doing so in the manuscript text, or remove these references and replace them with relevant current references. Any changes to the reference list should be mentioned in the rebuttal letter that accompanies your revised manuscript. If you need to cite a retracted article, indicate the article’s retracted status in the References list and also include a citation and full reference for the retraction notice.

**Response:** We corrected the issue where the doi codes or URL for four citations had a "." at the end, which made them unlinkable. We were unable to find the retracted paper. Of course, if there is one, I would like to correct it. Could you please let me know?

[7] Mao Y, Roberts S, Pagliaro S, Csikszentmihalyi M, Bonaiuto M. Optimal experience and optimal identity: a multinational study of the associations between flow and social identity. Front Psychol. 2016;7. doi:10.3389/fpsyg.2016.00067

[13] Panduro J, Ermidis G, Røddik L, Vigh-Larsen JF, Madsen EE, Larsen MN, et al. Physical performance and loading for six playing positions in elite female football: full-game, end-game, and peak periods. Scand J Med Sci Sports. 2022;1(Suppl. 1): 115-126. doi:10.1111/sms.13877

[22] Une N. The "Most Average Hitter" in 2020 is... In the Pacific League, the batting average, on-base percentage, and slugging percentage are one point behind the average. [in Japanese; Internet]. 2020; https://news.yahoo.co.jp/byline/unenatsuki/20201128-00209940 [Accessed 2 May 2023].

[23] Une N. The "most average hitter" in 2021 is... The average OPS of players who have reached the regular at-bat is .783 for Central League and .763 for Pacific League. [in Japanese; Internet]. 2021; https://news.yahoo.co.jp/byline/unenatsuki/20211209-00271866

[27] Lindsey GR. An Investigation of Strategies in Baseball. Ops Res. 1963;11: 477-501. doi:10.1287/opre.11.4.477

**Additional Editor Comments:**

Thank you for your responses. Some of the wording in lines 310-318 should be modified. Please consider the following re-write:

"However, we also considered that our data may not apply to and fit all game situations. For example, when the score is tied, the importance of performance increases in the ninth inning compared to the first inning [1]. This may lead batters to be more sensitive about their perception of the count, but pitchers may also show a similar tendency. Additionally, the difference in the number of times a batter is at the plate compared to the number of batters a pitcher faces might be associated with the difference in perception between batters and pitchers. That is, once a batter finishes an at-bat, there is an interval of eight batters before the next at-bat. In contrast, a pitcher faces all batters consecutively. The small number of times a batter is at the plate in a baseball game may lead to greater sensitivity for batters than for pitchers."

Please also consider whether this is the way you wanted to state this particular thing: "For example, when the score is tied, the importance of performance increases in the ninth inning compared to the first inning [1]." That sounds as if batters care more about their performance in the ninth inning compared to the first inning. Does this actually mean that expectations change in terms of what pitches might be thrown, or does it actually mean that batters are not as "tuned in" to the count in first inning?

**Response:** We apologize for our insufficient explanation. We consider that expectations have changed, as you pointed out. However, we think of it as an expectation for victory, not for pitch type. If the score is the same 1-0, the meaning of the situation is very different depending on the inning. For example, in the 1st inning, there are 9 chances to turn the game around; by contrast, in the 9th inning, there is only one chance to turn the game around. It is conceivable that batters and pitchers consider the 9th inning to be more important than the 1st inning. For this reason, we expect that the perception of advantage or disadvantage of batters and pitchers based on outs, balls, and strike counts will fluctuate more in the 9th inning than in the 1st inning. We added this explanation in Discussion section (page 18, lines 317-324):

“We also considered that our data could not apply to and fit all cases in the game situation. For example, when the score is tied, the importance of performance increases in the ninth inning than in the first inning [1]. In other words, expectations for victory have changed, depending on inning. If the score is the same, that is. 1–0, the meaning of the situation can be very different. For instance, in the first inning, there are nine chances to turn the game around; by contrast, in the ninth inning, there is only one chance to turn the game around. It is conceivable that batters and pitchers consider the ninth inning to be more important than the first inning. For this reason, we expect that the perception of advantage or disadvantage of batters and pitchers based on outs, balls, and strike counts will fluctuate more in the ninth inning than in the first.”

In addition, we have reconsidered the structure of the text and moved the following sentences (page 18, lines 309-314).

“We hypothesized that this was related to the differences in perception between batters and pitchers, which were in turn based on the difference in the number of plays. Specifically, once a batter is in at-bat, there is an interval of eight batters before the next at-bat as batting order. By contrast, a pitcher faces all batters continuously. The small amount of batting play in a baseball game may lead batters to be more sensitive.”
